# Impact of the Tip-to-Semiconductor Contact in the Electrical Characterization of Nanowires

**DOI:** 10.1021/acsomega.3c08729

**Published:** 2024-01-24

**Authors:** Juliane Koch, Lisa Liborius, Peter Kleinschmidt, Werner Prost, Nils Weimann, Thomas Hannappel

**Affiliations:** † Department of Mathematics and Natural Science, Institute for Physics, Fundamentals of Energy Materials, Ilmenau University of Technology, Ilmenau 98693, Germany; ‡ Components for High Frequency Electronics (BHE), University of Duisburg-Essen, Duisburg 47057, Germany

## Abstract

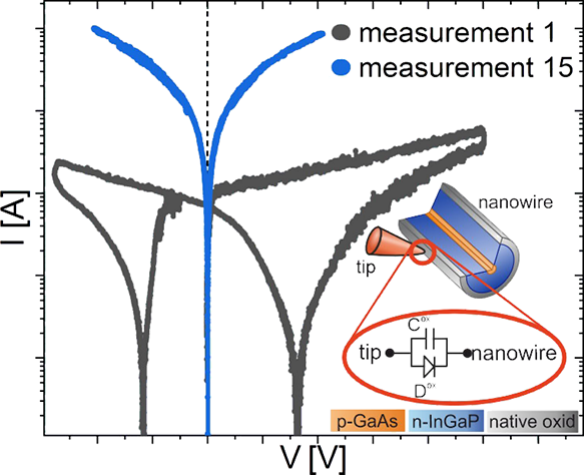

Well-defined semiconductor heterostructures are a basic requirement for the development of high-performance optoelectronic devices. In order to achieve the desired properties, a thorough study of the electrical behavior with a suitable spatial resolution is essential. For this, various sophisticated tip-based methods can be employed, such as conductive atomic force microscopy or multitip scanning tunneling microscopy (MT-STM). We demonstrate that in any tip-based measurement method, the tip-to-semiconductor contact is decisive for reliable and precise measurements and in interpreting the properties of the sample. For that, we used our ultrahigh-vacuum-based MT-STM coupled *in vacuo* to a reactor for the preparation of nanowires (NWs) with metal organic vapor phase epitaxy, and operated our MT-STM as a four-point nanoprober on III–V semiconductor NW heterostructures. We investigated a variety of upright, free-standing NWs with axial as well as coaxial heterostructures on the growth substrates. Our investigation reveals charging currents at the interface between the measuring tip and the semiconductor via native insulating oxide layers, which act as a metal–insulator–semiconductor capacitor with charging and discharging conditions in the operating voltage range. We analyze in detail the observed *I*–*V* characteristics and propose a strategy to achieve an optimized tip-to-semiconductor junction, which includes the influence of the native oxide layer on the overall electrical measurements. Our advanced experimental procedure enables a direct relation between the tip-to-NW junction and the electronic properties of as-grown (co)axial NWs providing precise guidance for all future tip-based investigations.

## Introduction

III–V semiconducting nanowire (NW) structures offer a great potential for high-performance (opto-)electronic devices such as LEDs,^1−5^ lasers,^6,7^ and heterobipolar transistors^8^ and for solar energy conversion devices such as solar cells^9−11^ or solar fuel devices.^12−15^ They can be engineered in a wide range of applications due to their tunable electronic band structure via the choice of material composition and doping profiles,^16^ e.g., for the integration of charge selective contacts,^17^ heterocontacts for tunnel junctions,^18^ or tunnel barriers.^8,19^ Due to the high surface-to-volume ratio, special attention must be given to their surface and interfacial junctions, e.g., in the case of coaxial heterostructures.^20^

For that, precise analysis techniques for the required (opto)electronic properties of the bottom-up prepared NW structures have been established in recent years. Of particular interest for novel structures are techniques that investigate the local current behavior and the associated material profiles and charge carrier distribution within the NWs. Hence, electrical measurements are required that separate the contact behavior from the transport properties of charge carriers in the NWs. In the case of epitaxially prepared structures, the four-point transmission line method (TLM)^21,22^ has been applied in the past also to NWs^23−25^ at the expense of a quite complex high-resolution fabrication technology. In addition, the spatial resolution when applying TLM is very low, in particular for NW structures, and the measurement of free-standing NWs is not possible at all, including the determination of the NW-to-substrate interface resistance. This can be remedied by tip-based measurement methods such as conductive atomic force microscopy (C-AFM)^26−28^ or multitip scanning tunneling microscopy (MT-STM).^25,29−31^ Only tip-based measurement methods provide suitable nanoscale resolution for electrical measurement; here, a clear definition of the contact behavior is a requirement for the interpretation of measurement results of tip-based measurement methods.

We investigated the contact behavior between the measuring tip-to-semiconducting NW junction with the help of an MT-STM and a built-in scanning electron microscope (SEM). By applying this method, detailed electrical investigations with high spatial resolution on NW structures are feasible.^25,29,30,32^ We focus on the influence of the tungsten tip-to-semiconductor junction on the overall electrical characteristics of the investigated samples.

We examined five different types of GaAs-based, upright standing NW structures on the growth substrate in axial and coaxial configuration for comparison. We prepared all samples via the vapor–liquid–solid (VLS) procedure. A unique experimental setup was used to study selected examples, including a state-of-the-art NW preparation by metalorganic vapor phase epitaxy (MOVPE), a contamination-free transfer from the MOVPE to the MT-STM in ultrahigh vacuum (UHV),^33^ and the four-point probe measurement setup of the MT-STM with fully 3D adjustable piezoelectric nanopositioners for each individual tip.^34^ By precise analysis of the recorded electrical MT-STM measurement data, we could clearly determine the contact behavior between the tungsten tip and the semiconducting NW and improve it in the next step. Our work provides a route toward well-defined direct contact probe-based measurements (i.e., MT-STM, C-AFM, scanning voltage microscopy (SVM),^35^ and scanning spreading resistance microscopy (SSRM))^36^ of electrical properties of semiconductor nanostructures.

## Results and Discussion

**Figure 1:**
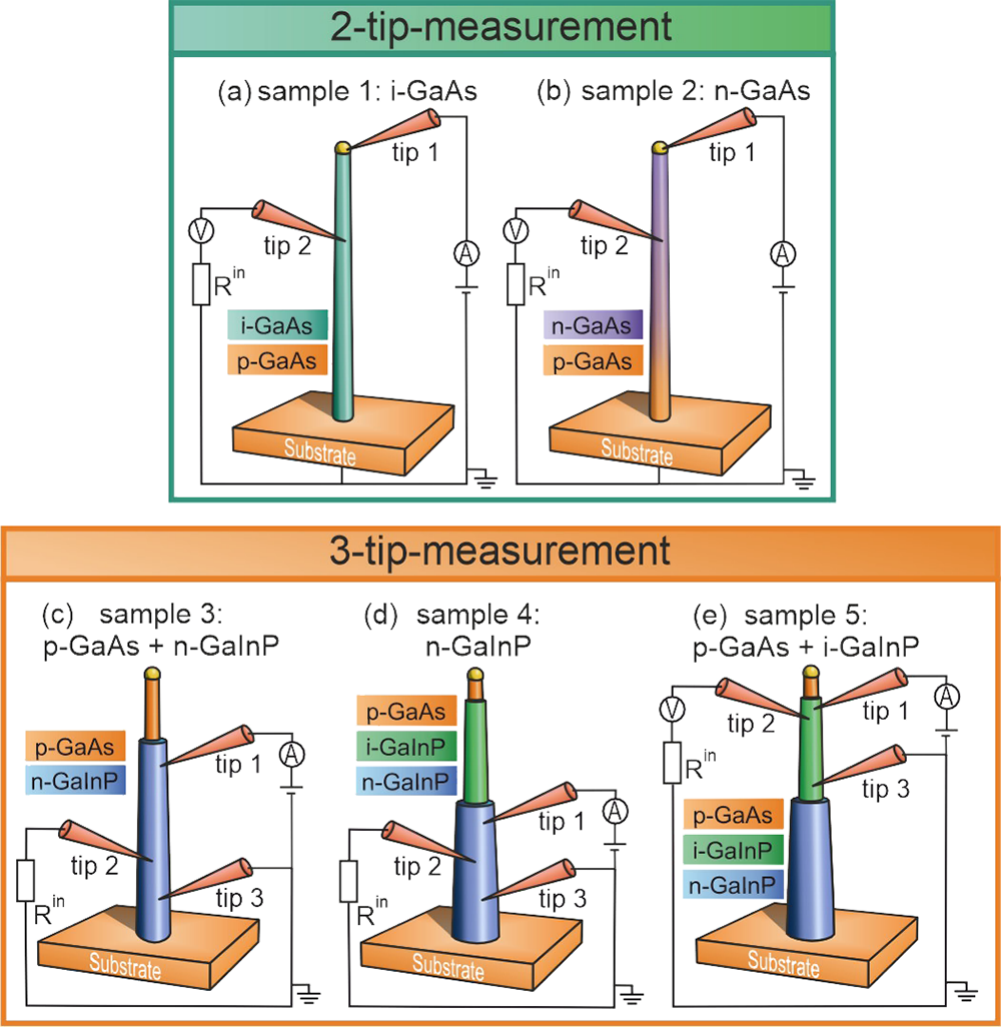
Schematic sketch of three-point MT-STM measurement principle. (a, b) two tungsten tips and one back contact and (c–e) three tungsten tips for different NW configurations.

In this section, we analyze the electrical behavior of the tip-to-semiconductor contact on MOVPE-grown GaAs-based NWs with different axial and radial structures, as shown in Figure 1, in detail, utilizing the MT-STM in a three-terminal measurement mode. In the case of a two-tip measurement setup, we used the substrate back contact as a third contact for passing current through the wire. In contrast, the substrate is not connected in the three-tip measurement setup. First, we determined the voltage behavior at the potential probe tip (tip 2) in relation to the current source tip (tip 1), at which we applied the voltage in all measurements. Then, we analyzed the measured voltage–applied voltage (*V*_m_–*V*_a_) and the measured current–measured voltage (*I*_m_–*V*_m_) characteristics with regard to the contact behavior using a n-GaInP NW as an example. Here and in the following, the index “m” corresponds to “measured” and “a” corresponds to “applied”. In analogy to the measurement setup, we created an equivalent circuit and discussed the influence of the measurement setup on the *I*–*V* characteristics in detail. Furthermore, we explain the change in contact behavior between the probe tip (tip 2) and the semiconductor with local oxide removal.

### Electron Beam-Induced Current (EBIC) Measurements

**Figure 2:**
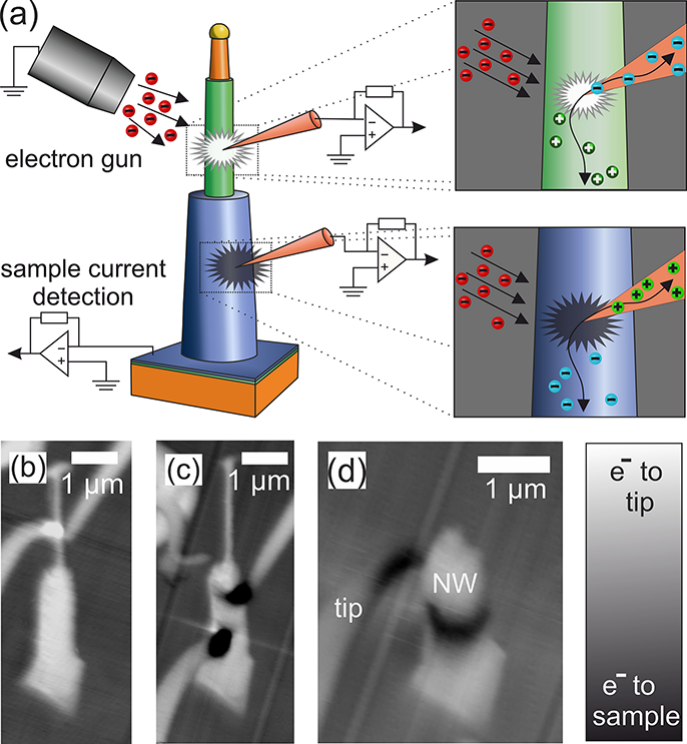
EBIC measurements at individual NWs: (a) schematic of charge-selective contacts between the tungsten tips and the semiconductor, (b) tip contacted at sample 5 (p-GaAs core/i-GaInP shell NW), (c) tip contacted at the n-GaInP shell of sample 4, and (d) tip contacting the n-GaInP shell at the backside of the NW of sample 4 exhibiting a black ring as a circular equipotential surface around the NW. Due to charge carrier diffusion through the highly conductive tungsten tip, a characteristic signal is already generated when the incoming electron beam hits the measuring tip near the charge-separating contact. EBIC signals confirm the positions of contact along the NW as well as the charge carrier selectivity at the tip-to-semiconductor junction, when a contact between the probe and the semiconducting NW is made.

In order to obtain a first indication of the properties of the tip-to-semiconductor contact, we carried out spatially resolved EBIC measurements at the NW. The incident electron beam of the built-in SEM on the sample generates electron–hole pairs locally in the NW,^37^ which can either recombine according to the charge carrier lifetimes, or electrons and holes can be separated at a charge-selective contact^17^ (e.g., metal–semiconductor (Schottky)-contact, metal–insulator–semiconductor (MIS)-contact, p–n-junction), where the electron–hole pairs can be separated and detected as a contribution to the sample current measured by the transimpedance amplifier, as depicted in Figure 2. In an axial NW scan, we can identify different heterogeneous regions, as illustrated in Figure 2a: the tungsten probes or the NW itself can cause a bright EBIC signal representing a region where a smaller amount of electrons arrives at the substrate; accordingly, a larger electron flow to the substrate appears as a dark spot. At the direct connection of the measuring tips (tip 1 and 2) with the NW of sample 5 (p-GaAs core and i-GaInP shell), which is shown in Figure 2b, a bright signal becomes visible at the contact point. From this signal, we conclude that the type of contact causes a selective transport of electrons to the measuring tip, i.e., we detected an increased electron current from the core consisting of p-GaAs and surrounded by a thin i-GaInP shell toward the tungsten measuring tip. In contrast, in Figure 2c, a dark signal appears, when we brought the tip in contact with the n-GaInP shell of sample 4, which depicts a charge carrier selective contact^17^ indicated by a band bending between the tip and the n-doped semiconductor that causes an increased electron current to the substrate.

**Figure 3:**
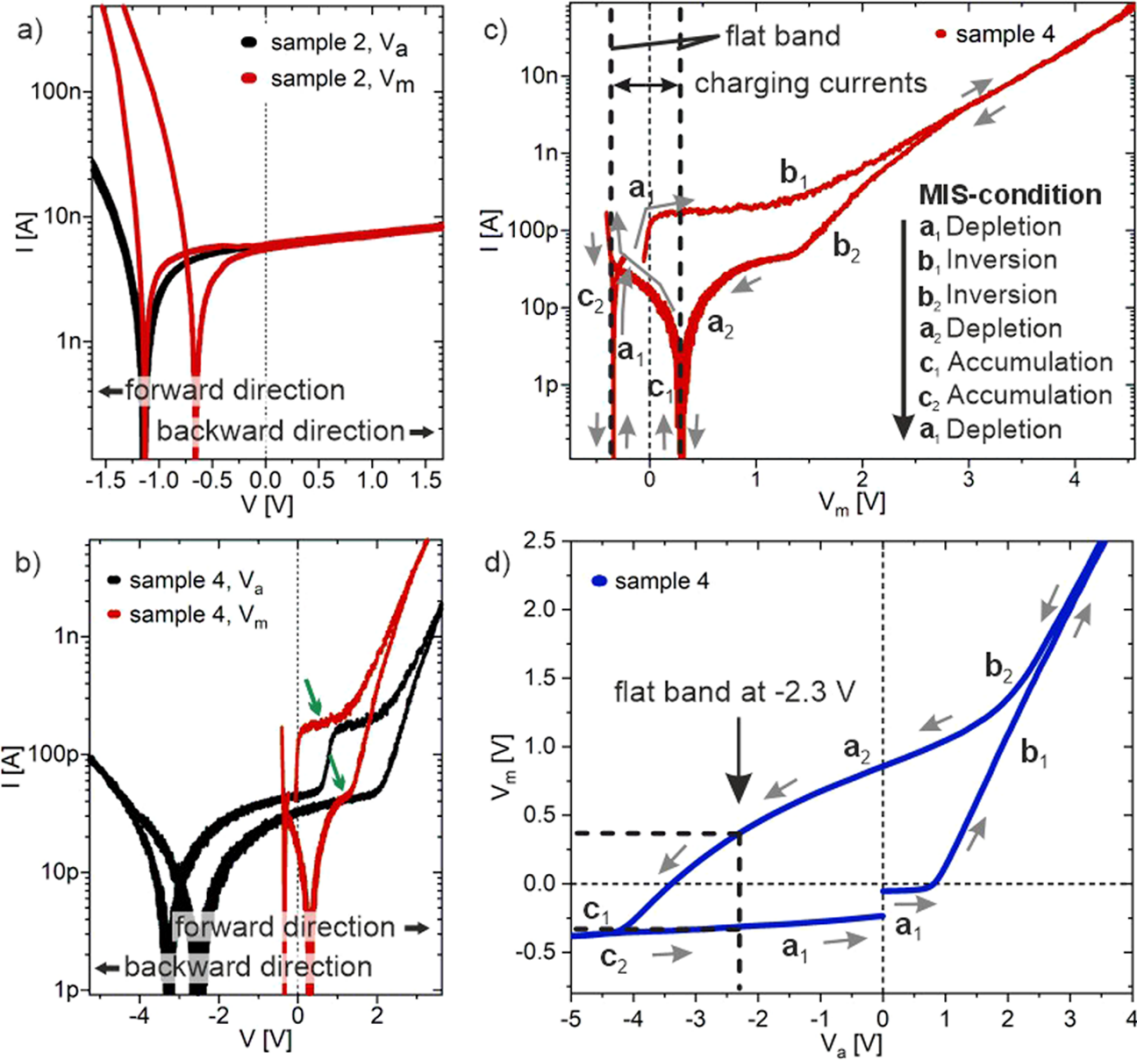
MT-STM measurements: (a–c) *I*–*V* characteristics of samples 2 and 4 with applied (*V*_a_, black) and measured (*V*_m_, red) voltages at tip 2. (a) First measurement after contact of sample 2 and (b) third measurement after contact of sample 4. Green arrows highlight the comparatively flat curve section. n-GaAs and n-GaInP NWs show hysteresis in the measured current in comparison between forward and backward directions over the voltage range. (c) *I*_m_–*V*_m_ curve of sample 4 extracted from the plot in (b) for detailed analysis. (d) *V*_m_–*V*_a_ plot of sample 4. The direction of the applied potential in plots (c) and (d) is symbolized by gray arrows: starting at 0 V, passing through positive potentials, crossing 0 V to negative potentials, and back to 0 V. MIS conditions are marked with a, b, and c.

In addition to the spot at the tip-to-semiconductor point contact, a black, ring-shaped signal appeared around the NW at the tip-NW contact. This became particularly obvious when we were contacting the semiconductor, in this case, the n-GaInP shell of sample 4, at the NW facing away from the SEM gun (“backside” of the NW), as shown in Figure 2d. From this, we can conclude that a circular equipotential surface forms around the shell at the same height at the NW, where the contact is made. This shows that the measuring probes do not have to be arranged in a line for reliable measurement; only the position along the NW is relevant but not the exact contact location around the wire. In order to analyze the type of contact more precisely and to distinguish whether a Schottky contact or an MIS contact is present, electrical characterization of the contact behavior was carried out by different measurement series: first, we recorded the *I*_m_–*V*_a_ dependency, black curves in Figure 3, and second, the *I*_m_–*V*_m_ behavior, red curves in Figure 3. In addition, we show the *V*_m_–*V*_a_ dependency in Figure 3d.

### Contact Behavior

**Figure 4:**
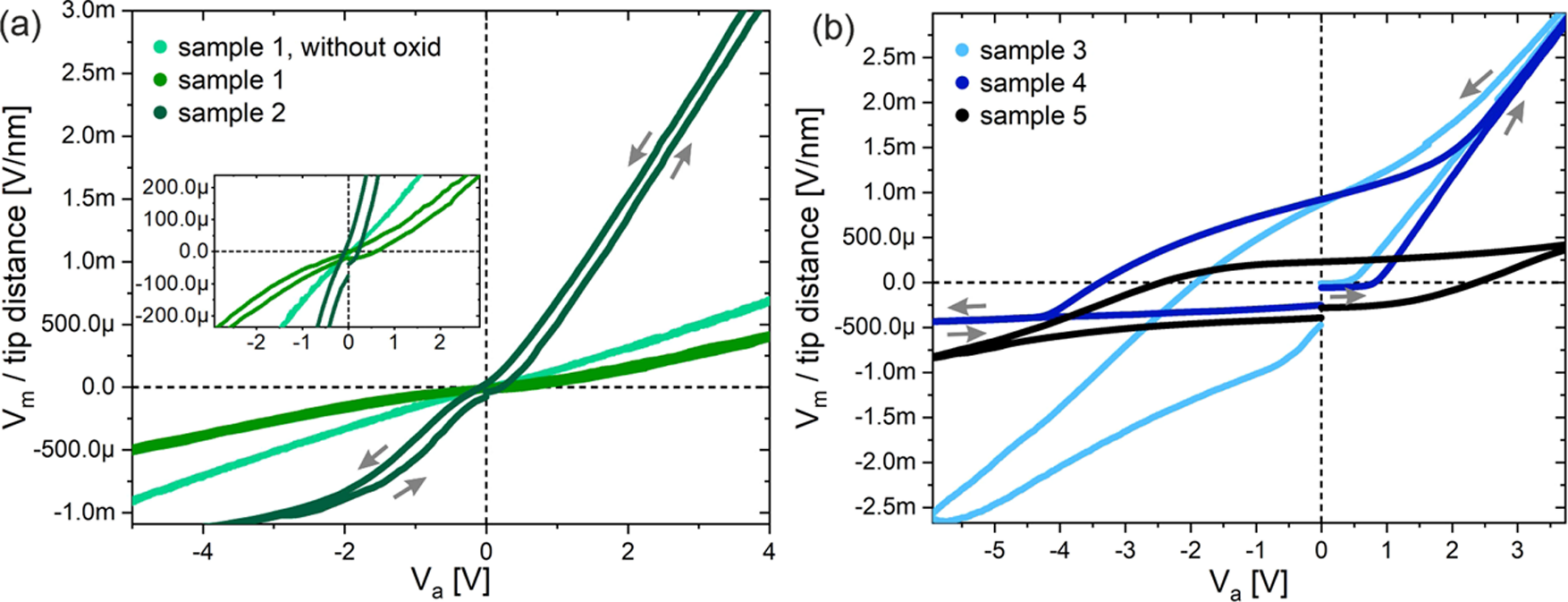
MT-STM measurements of individual NWs are represented in *V*_m_ versus *V*_a_ curves. Measurements according to Figure 1: (a) two-tip measurements, intrinsic NWs: sample 1 with and without native oxide and sample 2; inset for close up; (b) three-tip measurements, samples 3–5. All oxidized samples showed hysteresis in the measurements. Gray arrows symbolize the direction of changes of the applied voltages: starting at 0 V, passing through positive potentials, running across 0 V to negative potentials, and back to 0 V.

In order to determine the general influence of the potential probe tip and its contact properties to the semiconductor, we took *I*–*V* measurements in the forward and backward directions with two tungsten tips and used the back (substrate) contact as the third terminal for analysis, as depicted in Figure 1a,b. For the current source tip (tip 1), a metal-to-metal contact is expected between the tungsten tip and the gold particle, which we applied as a catalyst for the NW growth; here, the gold particle forms a Schottky-like contact to the semiconducting NW. To obtain a current flow, we used the back contact of the sample as a large area contact. During the measurements, we ramped the voltage up from 0 to +6 V, then down from +6 to −6 V, and up again from −6 to 0 V. It should be noted that the orientation of the voltage axis in Figure 3 is according to the measurement setups in Figure 1. For the corresponding *I*–*V* characteristics of sample 2, the direction of the electron current flow from n to p (the actual backward direction of the p–n junction) corresponds to positive voltage values in Figure 3a. First, we measured and plotted the current against the applied voltage between tip 1 and the back contact without taking tip 2 into account (black curve) and without any difference while ramping up and down. This two-point measurement reflects the typical behavior of a diode, when superposing various ohmic resistances connected in series of the entire experimental setup as well as selective transport via heterocontacts such as Schottky contacts.^25^ It shows a clear shift of the *I*–*V* curve along the voltage axis, so that the current minimum is at approximately *V* = −1.4 V owing to the presence of a thermoelectric voltage and already indicating high temperature gradients in the spatial range around the contacts (see below). Due to the series resistance, the *I*–*V* curves for the two-point measurement (*I*_m_–*V*_a_) exhibit a lower slope around the zero crossing than for the three-point measurement (*I*_m_–*V*_m_), Figure 3 a, red curve. Here, the measured current between tip 1 and the back contact is plotted versus the measured voltage at the potential probe tip (tip 2) with respect to the back contact. With this three-terminal measurement method, various series and contact resistances between tip 1 and the back contact can be neglected. However, a hysteresis with two current minima appeared in the recorded *I*–*V* data when the voltage was ramped up and down. This effect can be attributed to a component that includes a chargeable capacitance. Four rectifying (charge selective) contacts are conceivable in the present design of measuring probes. The contact at the current source tip 1 can be excluded, since it only entails the metal-to-metal contact to the gold droplet. This also applies to the Schottky contact between the gold drop and the NW as well as the large area back contact. Therefore, we considered the junction between the potential probe tip and the NW as well as the junction from the wire to the substrate. The contact between the wire and the substrate can also be ruled out as the cause of the hysteresis, having in mind that we observed a linear, ohmic response between the applied voltage at tip 1 and the measured voltage at tip 2 for a UHV-transferred NW, i.e., a NW without surface oxidation, as depicted in Figure 4a for sample 1. Accordingly, only the contact between the probe tip 2 and the NW can cause the hysteresis in the measured voltage and thus in the measured *I*–*V* characteristic.

Next, we positioned the current source tips on the side of the NW instead of the gold particle and the back contact as illustrated in Figure 1c–e. In this measurement setup, we observed a contact behavior that changed the *I*–*V* characteristic curve of the measured current versus the applied voltage (*I*_m_–*V*_a_), as shown in Figure 3b, black curves for sample 4. Here, the total current is obviously influenced by the contact behavior between the tungsten current probe and the semiconducting NW and shows a clear hysteresis with two current minima in the *I*–*V* characteristics. The respective junctions of both of the two measuring tips to the semiconducting NW are conceivable as the reason for the observed hysteresis. Which measuring tip provides the dominant contribution to the hysteresis could depend on the shape of the tip and thus details of the material composition at the contact area, doping profiles, inhomogeneities, contaminants, etc. Thus, it becomes clear that in addition to the influence of the contact between the potential probe tip 2 and the NW, which was explained before and shown in Figure 3a, the contacts of the current source tips to the NW also make a clear considerable contribution to the recorded *I*–*V* characteristics.

As expected in a two-point-probe measurement and already explained for Figure 3a, the black *I*–*V* curve exhibited the current increase at higher voltage values in comparison to the red curves due to the superposition of various resistances. The red curves describe the source current (tip 1) plotted against the probe potential measured at tip 2. Since the *I*_m_–*V*_m_ curve in Figure 3b shows a similar course compared to the *I*_m_–*V*_a_ curve, we conclude that the contact at the current source tips (tip 1 and tip 3) largely determine the *I*_m_–*V*_m_ characteristics. There is a clear formation of a comparatively flat section between two sections with larger slopes in the curve, marked with green arrows. This applies to ramping up and down. The details of the *I*_m_–*V*_m_ (red) curve of Figure 3b become more visible in Figure 3c. It becomes obvious that it does not follow a typical diode behavior. This drastic deviation of the actual expected diode characteristics can be explained by the superposition of the *I*–*V* characteristics with a MIS contact forming an MIS-like tunnel diode.^38^ If the *I*–*V* characteristics is compared with the literature,^39^ in which accumulation, depletion, and weak and strong inversions are described as MIS conditions, the respective MIS conditions can be identified with the characteristics of curves *I*_m_–*V*_m_ (Figure 3c) and *V*_m_–*V*_a_ (Figure 3d). Due to charging and discharging effects caused by the band bending as well as defect states,^40,41^ a strong hysteresis also occurs here. In the case of an n-GaInP NW (sample 4), the typical features of an MIS structure are passed through starting with a charge carrier depletion in the semiconductor marked with a_1_ at an applied voltage of 0 V. When a positive voltage is applied to the semiconductor, the energy bands are shifted with respect to each other so that an inversion state of charge carriers occurs in the semiconductor (b_1_). After reaching a maximum positive voltage of +6 V, the voltage is ramped down again. The typical MIS states of inversion (b_2_) at high voltages and depletion (a_2_) at low voltages are passed through when the MIS capacitor is discharged. This can be seen especially in the *V*_m_–*V*_a_ plot, where a positive voltage of 0.85 V is measured at a 0 V applied voltage. With negative voltages, the flat band state is reached at −2.3 V, followed by accumulation of charge carriers in the semiconductor (c_1_). Ramping up the voltage from −6 to 0 V, the accumulation state (c_2_) occurs, followed by passing through the flat band state again at an applied voltage of −2.3 V and, finally, returning to the depletion state (a_1_).

Due to the MIS contact behavior, there are charging currents during the actual transition through the flat band state resulting in two minima in the *I*–*V* characteristics and thus two flat band states related to the measured voltage at 0.39 and −0.33 V. Such charging currents had already been observed for Schottky contacts in connection with oxide thin films by Splith et al.^42^ and Ahn et al.^43^ Due to the hysteresis and the associated stored charge carriers (a_2_ → c_1_: positive/holes or c_2_ → a_1_: negative/electrons) as well as their different mobilities, the absolute values of the measured voltages differ (a_2_ → c_1_: 0.39 V and c_2_ → a_1_: −0.33 V).

In order to gain more insight into the effects of the contact behavior of the potential probe tip on the characteristic curves, we compared the measured voltage at tip 2 with the applied voltage at tip 1 for all samples. For a better comparison, we normalized the measured potentials to the distance between the measuring tips 1 and 2 in Figure 4. Here, we ramped the applied voltage up and down starting from 0 V as illustrated in Figures 4 within 120 s for each sample.

Without oxide, there is an almost linear dependence between the applied and measured voltage. Based on this, we can rule out the possibility that the measuring tips oxidize again in the UHV after sputtering, and therefore, the pure measuring tip surface does not cause the hysteresis. Since the work function of tungsten is approximately equal to that of the semiconductor, the slope at positive applied voltages is equal to the slope at negative values. If the semiconductor is p- or n-doped (compare samples 1 and 2), the slopes differ for the positive or negative voltage range. This fact applies, in particular, to sample 2. It can be explained by tip-induced band bending theoretically described by Voigtländer et al.^44^ During the establishment of a contact, a contact potential between the tungsten tip and the NW is formed. The application of a voltage leads to a significant shift of the Fermi energy of the metal and, accordingly, band bending in the semiconductor.

The plot of the measured voltage versus the applied voltage in the different directions (changes up and downward) exhibits a hysteresis in dependence on the direction of changes. This also applies to all oxidized NWs as charging effects can be generated in the up and down directions because of the present oxide, ending up in the observed hysteresis. When we compare the different samples, we can see that the charging effects are significantly dependent on the used measurement setup (two-tip vs three-tip). Due to the different contacts (two current source tips to the semiconductor and potential probe tip to the semiconductor) and their superposing characteristics, the samples with three measuring tips at the NW show a more distinct and complex development of the hysteresis. With the three measuring probes at the NW, the individual contact characteristics of the measuring tips are superposing, whereby the effect of the potential probe tip is less than that with the current source tips. Especially, the n-doped GaInP-based samples, such as sample 4 (n-GaInP), exhibit strong hysteresis characteristics.

### Equivalent Circuit of Tip-to-NW Junction

**Figure 5:**
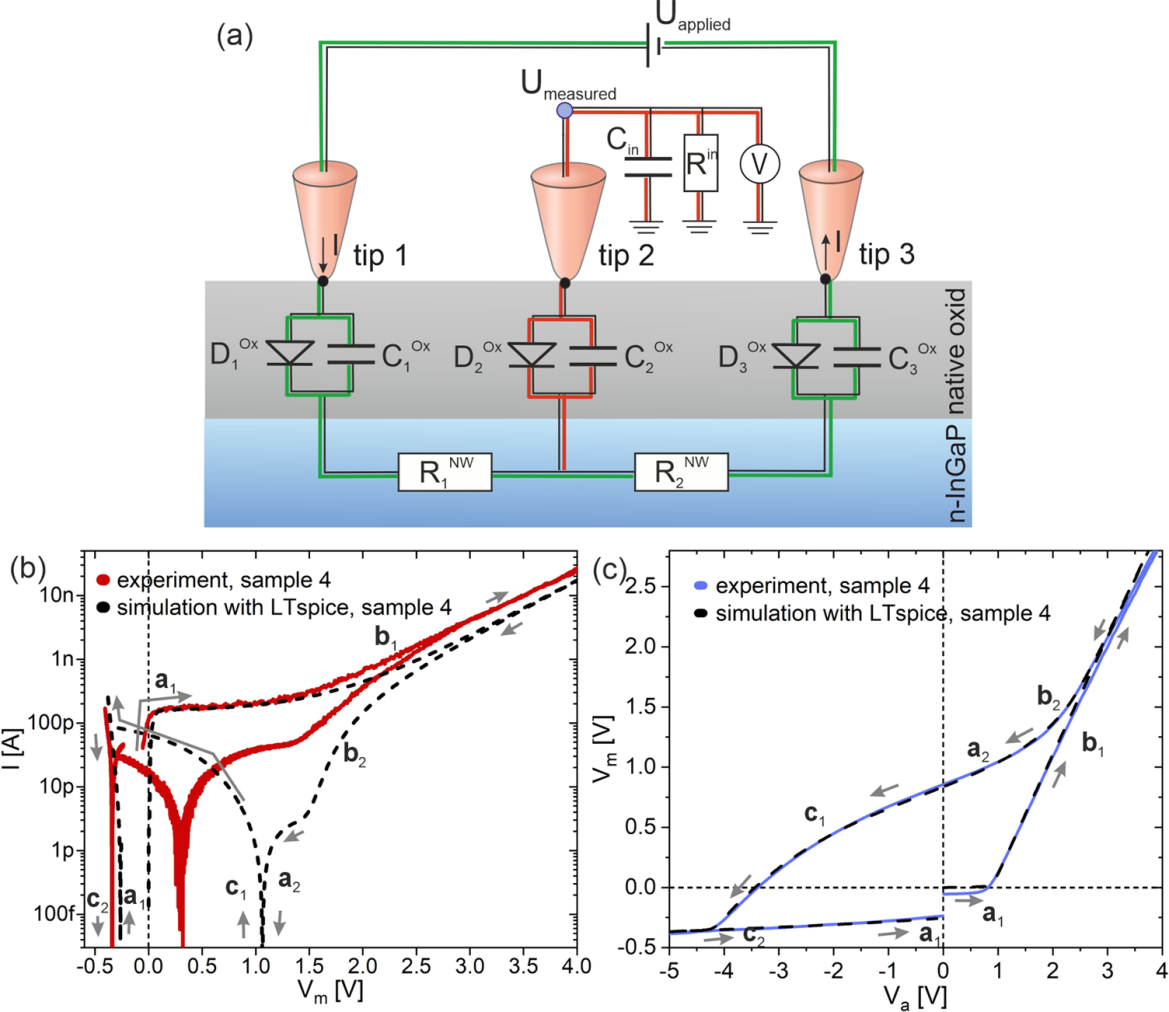
(a) Simulation of the equivalent circuit of the measurement setup. The current circuit is depicted with green lines, and the potential measurement part with red lines. The potential measuring probe has a high input resistance *R*_in_ of 10 MΩ with a parallel input capacitance *C*_in_. Each contact between the tungsten tips and the NW can be described by a diode D_i_^ox^ and a capacitor C_i_^ox^ connected in parallel. The specific resistance of the NW represented by two resistors R_i_^NW^; (b) simulation of the measurement setup of sample 4 plotted in a dotted black *I*_m_–*V*_m_ curve; (c) dotted black *V*_m_–*V*_a_ curve. For comparison, the experimentally recorded curves are displayed in color. The direction of changes in the applied potential is symbolized by gray arrows: starting at 0 V, passing through positive potentials, running across 0 V to negative potentials, and back to 0 V.

In Figure 5 we describe the measurement setup by an equivalent circuit consisting of several diodes and capacitors for the tip-semiconductor junctions, the bulk resistances of the NWs between the measuring tips, and the input resistance for the potential measurement.

As we have explained in the EBIC measurements, each measuring tip forms an M(I)S contact, which acts as a charge carrier selective contact and can be modeled as a Schottky or an MIS diode with specific characteristics and values essentially influenced by the thickness of the oxide layer. In the circuit of Figure 5a, a capacitor *C_i_*^ox^ (*i* = 1,2,3) is connected in parallel to the diodes, in order to represent the chargeable electronic states of the oxide layer. For the potential measurement at tip 2, we connected a voltage meter in parallel with a high input resistance *R*_in_ of 10 TΩ and an input capacitance *C*_in_.

In order to estimate the influence of the measuring setup (instruments and wire connections) on the measured values, we studied the current–voltage behavior, considering a worst-case scenario in which the NW resistance is very high compared to the input resistance of the potential measuring device. For that, we used values of 20 GΩ for the NW resistance and 10 TΩ for the input resistance. In the recorded *I*–*V* characteristic, a hysteresis occurred throughout the voltage range due to the instrumental setup. However, this hysteresis was much smaller than the hysteresis observed in the NW measurements. The difference in hysteresis was at least an order of magnitude smaller when current values were compared at given voltages. Furthermore, this hysteresis exhibited a constant offset over the entire voltage range in both directions of voltage changes. This worst-case scenario already shows that the impact of the setup on the real characteristic curve can be expressed by a capacitor *C*_in_ parallel to the input resistance *R*_in_ in the equivalent circuit. Because of the small impact at the real *I*–*V* curves, the capacitance *C*_in_ can be estimated in the aF range.

Based on the fact that real NW resistances have significantly lower values, we can conclude that the impact of the setup on the *I*–*V* characteristics is negligible. The NWs used in these investigations have resistances in the kΩ to maximum MΩ range.^29,30,32^ The previously used exemplary sample (n-GaInP) from Figure 1d, which originates from a set of samples with the same growth conditions and doping profiles already shown in Liborius et al.,^24^ can be estimated with a resistance *R*_1,2_^NW^ of 50 Ω/μm. This demonstrates that the real values of the NW resistances are significantly below those used in the worst-case scenario. Therefore, the measured hysteresis with the characteristics *I*_m_–*V*_m_ and *V*_m_–*V*_a_ (see Figure 3c,d) reflects the contact characteristics between the measuring tip and the NW contact.

Next, we performed a simulation of the equivalent circuit for sample 4 as shown in Figure 5 by applying the program LTspice XVII. A Schottky diode was used as the basis for the electrical description of each tip, in which the saturation current density and reverse breakdown voltage were varied until simulation and measurement were in good agreement. The exact parameters are given in the Supporting Information. Since each probe may differ in its shape and the associated contact area with the NW, the contact behavior varies in its characteristics, especially regarding saturation current, reverse breakdown voltage, and current at the breakdown voltage. Therefore, the measured current is determined by the contact behavior of tip 1 through diode D_1_^ox^ and the capacitor C_1_^ox^ as well as tip 3 by diode D_3_^ox^ and the capacitor C_3_^ox^. For tip 2, a strong reduction or even the absence of current through the probe is to be expected due to the high input resistance *R*_in_ of 10 TΩ. Therefore, the associated impact of the NW-to-tip contact behavior can be assumed to be smaller in comparison to the current source tips. The used values for the simulation are available in the Supporting Information.

The simulated curves of Figure 5b,c, displayed with dashed lines, are in agreement with the measured curves. The curves (*V*_m_–*V*_a_) and (*I*_m_–*V*_m_) exhibit hysteresis which is related to the MIS-states, marked with a, b, and c, comparable to those in Figure 3c,d. The contact potential was not taken into account in the simulation; therefore, the measured voltage is 0 V, equal to the applied voltage. With an applied voltage of 0 V (*V*_a_ = 0 V), one simulated flat band state of the (*V*_m_–*V*_a_) plot is at *V*_m_ = 0.84 V and the second state is at −0.25 V, while the real measured voltage at *V*_a_ = 0 V (see *V*_m_ at *V*_a_ = 0 V in Figure 3d) also takes similar values: 0.86 and −0.24 V. A slight discrepancy can be seen in the *I*_m_–*V*_m_ plot mainly under the first flat band condition, which could be explained by deviating diode parameters of the simulation compared to the experiment. The thermoelectric effect introduces an additional dependence of the electric potential on the temperature and therefore on the dissipated power. However, we based the simulation on the assumption that the diode parameters determined using the (*V*_m_–*V*_a_) plot are constant over the entire voltage range and are not temperature-dependent. Accordingly, a discrepancy occurs as a shift along the voltage axis in the (*I*_m_–*V*_m_) diagram compared to the measurement. Because of the high input resistance of 10 TΩ, it can be assumed that no current flows via the potential probe (tip 2). Hence, dissipation should be negligible at this point, but the exact temperature distribution along the NW is not known, and therefore, we cannot model thermal diffusion and its influence on the electrical current. Especially in the case of chargeable electronic states, thermal diffusion can lead to an additional contribution to charge currents and thus a shift of the flat band states along the voltage axis. Overall, the characteristic curves correlate quite well, showing that the hysteresis caused is due to the presence of the oxide layer between the tungsten tips and the semiconducting NW and hence the introduction of an additional capacitance.

By varying the capacitances as well as the diode parameters of the individual contacts in the model, it turned out that the best agreement between the measurement and the simulation can be found with C_1_^ox^ = 1 pF, C_2_^ox^ = 1 pF and C_3_^ox^ = 800 pF for the selected sample 4. This also reflects the behavior already shown. There is a superposition of the individual contacts of the measuring tips on the *I*–*V* characteristics, whereby the impact of the potential probe tip (tip 2) is less than of the current source tips (tips 1 and 3).

### Local Oxide Removal

The contact behavior shown before reveals the effects of the MIS contact between the measuring tip and the NW on the overall *I*–*V* characteristics in the entire voltage range. The desired electrical NW characterization (e.g., the resistance behavior of the NW or the mechanism of charge carrier transport along the NW) interferes with the electrical properties of the MIS contact, which complicates the evaluation of the addressed parameters of investigation. It is therefore necessary to modify the contact behavior between the measuring tips and the sample such that it plays a minor role or can be neglected.

**Figure 6:**
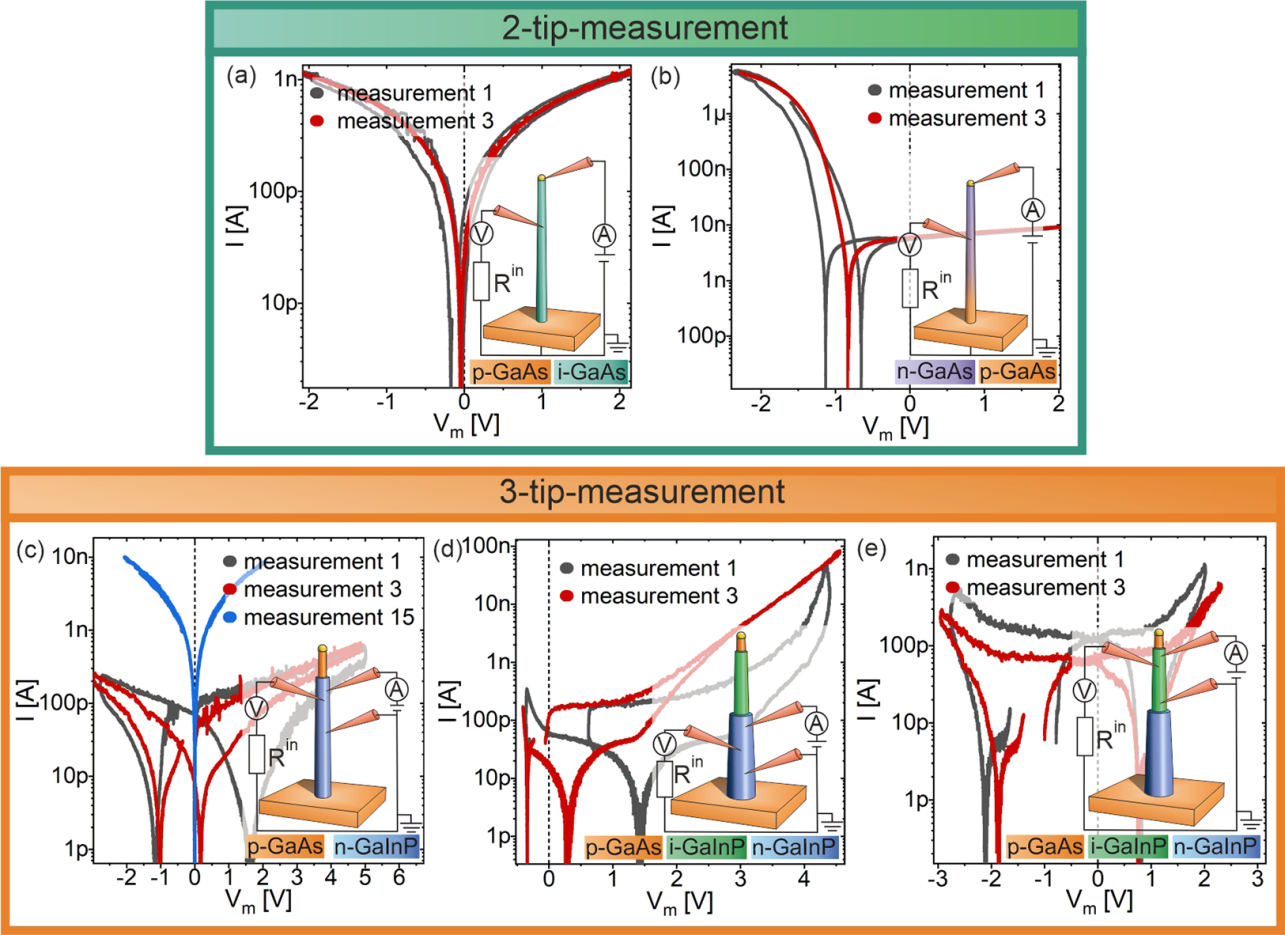
*I*–*V* characteristics for all samples: (a, b) two tungsten tips and one back contact: (a) sample 1 and (b) sample 2; and (c–e) three tungsten tips for different NW-configurations: (c) sample 3, (d) sample 4, and (e) sample 5. Measurements at first contact are displayed in black curves. The third measurement is displayed in red. The blue curve shows the 15th measurement without changing the contact area.

Figures 6a–e shows the recorded *I*–*V* curves associated with the samples 1–5 already depicted in Figure 1. The black curves represent the measurement after the first contact between the measuring tips and the NWs. All samples exhibit hysteresis, which can be described in analogy to Figure 3.

The third measurement at the same contact area at the NW is also displayed in Figure 6a–e as red curves. For all measurements, it becomes obvious that the *I*–*V* curves change with the number of measurements: the strength of the previously occurring hysteresis becomes weaker, while the rate at which the hysteresis changes depends on the material. The absolute values of the measured voltages of the two flat band states become smaller per measurement and approach each other. For the pure GaAs samples, the influence becomes sufficiently small after only three measurements; a hysteresis is no more visible. In contrast, the GaInP-based samples exhibit only a slightly smaller hysteresis in the third measurement after tip-to-semiconductor contact and thus a clear superposition of electrical properties of the tip-to-NW contact in the *I*–*V* characteristics. In the case of sample 3, for example, no hysteresis can be seen in the recorded *I*–*V* curve until after the 15th measurement. In Figure 6d,e, this behavior is also to be expected, but the measurement series was terminated after the fourth measurement in each case. The slow degradation of the native oxide layer also demonstrates that GaInP-based NWs can store a large amount of charge carriers and may be suitable for possible applications as NW capacitors.

Due to the small area of the tip-to-NW contacts, we can assume that there are high local current densities at the contacts. Depending on the material compositions, the induced current as well as the specific NW resistivity cause local heating of the NW contact obviously, resulting in local oxide removal and improvement of the tip-to-NW contact.

### Guide for reliable tip-based *I*–*V* measurements

We have shown that the measurement of tip-based *I*–*V* curves on semiconducting NWs can be complex and challenging due to the presence of intermediate oxide layers, leading to charging and discharging scenarios during the variation of current and voltage. We also showed that the oxide layers at the tip contact can be removed. Due to high current densities at the contact, thermal energy is applied leading to the removal of the native oxides. This process can be observed by successive reduction and, finally, by the absence of hysteresis. With this knowledge, we suggest a general strategy for a reliable and accurate tip-based *I*–*V* measurement. In order to establish a well-defined contact between the measuring tip and sample, the following procedure must be performed:

The semiconductor oxide shall be removed by repeated *I*–*V* measurement cycles leading to improved contacts between the measuring tips and the sample toward a Schottky-like, metal–semiconductor contact. This procedure shall be carried out (i) until no voltage offset between measured voltage *V*_m_ and applied voltage *V*_a_ remains, depicted in a (*V*_m_–*V*_a_) diagram, (ii) until no hysteresis, i.e., no charging and discharging effects, can be observed in (*V*_m_–*V*_a_) as well as (*I*_m_–*V*_m_) diagrams, and (iii) only one flat band condition in the (*I*–*V*) diagrams is seen. By implementation of this strategy, accurate and reliable tip-based *I*–*V* measurements can be obtained for semiconducting materials, overcoming the challenges posed by charging and discharging effects caused by oxide layers.

## Conclusions

For a general assessment and explanation of tip-based *I*–*V* measurements on semiconductor nanomaterials, we have examined two- and three-point measurements at individual III–V-nanowire (NW) structures utilizing a UHV-based multiprobe STM. In particular, we scrutinized the complex tip-to-semiconductor contacts on different examples of axial and radial GaAs/GaInP NW structures either covered with native oxides or without oxides in UHV. The determination of the electronic and material properties between the tungsten tips and the semiconducting NWs succeeded by the analysis of the *I*–*V* curves, specifically, by the evaluation of the dependence of the applied voltages as well as of the measured current on the measured potential. Charging and discharging cycles during the measurements lead to hysteresis in the *I*–*V* characteristics, in dependence of the specific NW properties. GaInP-based NW-structures, for instance, show significantly stronger charging behavior than GaAs-based ones. We explained that the observed hysteresis is attributed to the presence of native oxide layers between the measuring tips and the semiconductor materials, resulting in an MIS-like contact. We modeled the characteristics of the tip-to-NW contact by an equivalent circuit, based on the superposition of resistors, diodes, and capacitors for each sample, and described Schottky-like contacts in parallel with capacitors to represent the chargeable electronic states that originate from the native oxides on the NWs.

Reliable *I*–*V* measurements, free of artifacts, were achieved by the repeated application of *I*–*V* measurements, which is accompanied by local heating between the measuring tips and the semiconductor and a concomitant removal of the oxide layer. We suggest this procedure as a general routine that should be complied with to identify and ensure a reliable, oxide-free, and tip-based *I*–*V* measurement of semiconductor nanostructures.

## Experimental Section/Methods

### MOVPE Sample Preparation

For our studies, we prepared six different GaAs- and GaInP-based NW structures in a horizontal low-pressure Aixtron AIX 200 MOVPE reactor with H_2_ as the carrier gas at 50 mbar. In all cases, we used p-doped GaAs (111) type B substrates. A detailed illustration of the prepared structures is illustrated in Figure 1a–e, in which for all NW structures, a p-doped GaAs core was grown applying the VLS method and using gold particle catalysts of approximately 100 nm as a growth seed. Further growth details are reported elsewhere.^32^ The obtained p-doping level has been estimated at *N*_A_ = 2 × 10^18^ cm^–3^.^29^ For sample 2, the p-type doping was switched to n-type applying tetraethyltin (TESn) to reach a doping level of *N*_D_ = 2 × 10^17^ cm^–3^.^30^ The samples 3–5 are realized by the core–shell growth technique providing a GaInP shell grown at 650 °C.^9^ The doping level of the n-GaInP shell using ditertiarybutylsilane (DitBuSi) as n-dopant can be estimated to *N*_D_ = 3 × 10^18^ cm^–3^.^24^ Access to the inner core of the core–shell NWs of sample 3 and 5 is realized by selectively chemically wet-etching the top section of the GaInP shells by using hydrochloric acid.^24^ Thus, the p-doped NW core with a very thin inner shell is exposed at the top of the NW, providing access for positioning of the multitip scanning probes of the MT-STM on the NW inner part. The possible influence of a native oxide layer on the MT-STM characterization was evaluated with sample 1. This sample 1 was transferred contamination-(oxide-)free from MOVPE to the MT-STM in UHV.^33^ Later, the sample was exposed to air (and thereby covered by a native oxide layer) and investigated again. All other samples were exposed to ambient air and hence covered with native oxide directly after the growth process before loading into the MT-STM. The native oxide layer can be estimated at 2–3 nm according to the literature.^45,46^

### MT-STM Measurement

An individual, free-standing NW can be electrically examined nondestructively with a TetraProbe MT-STM from mProbes, whereby the UHV chamber and the measuring electronics are from Createc. In addition, an ECLIPSE+ SEM column from Orsay Physics can be used to determine the contact positions of the measuring tips to the NW. Depending on the desired effects, a common secondary electron detector (SED) mode or a sample current measurement mode can be employed. The sample current mode, see Figure 2, allows a visualization of charge-selective contacts with high resolution: the 25 keV electron beam of the SEM can generate mobile charge carriers, and due to the charge-selective contact, a current results, which is measured by a transimpedance amplifier.

For the electrical investigations, three terminal measurements were performed with three tungsten probes or with two tungsten probes and a back contact, as depicted in Figure 1. All measuring tips were sputtered with argon ions for 45 min, under 10 mA, 2.5 keV, and 6 × 10^–6^ mbar pressure before use in the UHV and thus cleaned of oxide. Here, individual control of the tungsten probes is achieved by piezo elements, so that precise positioning of the contacts along the wire is feasible.^34^ Subsequently, an electric current was induced through the NW by applying a voltage between measuring probes 1 and 3 or the back contact. For the potential measurements, another measuring tip was contacted spatially between the current measuring probes on the NW. This setup should exclude contact and series resistances from the measurement, which is crucial for accessing charge separating junctions, such as p–n or p–i–n junctions. For each measurement probe, it is possible to select between high impedance voltage and low impedance current measurement by means of a transimpedance amplifier and specially designed electronics.^47^ As a result, the potential difference between the potential probe tips can be determined to be almost current-free. All MT-STM measurements were carried out with the samples in UHV and at room temperature. In this work, six different samples with various NW configurations are investigated, which are shown in Figure 1. Each measurement was recorded in the forward and reverse current direction. The SEM is blanked during the measurement to prevent a contribution from the SEM-induced current.

## Supporting Information Available

Simulation parameters (PDF)

## ABBREVIATIONS

MT-STM: multitip scanning tunnelling microscope
SEM: scanning electron microscope
EBIC: electron-beam induced current
MOVPE: metalorganic vapor phase epitaxy
NW: nanowire
*I*_m_: *I*_measured_
*V*_m_: *V*_measured_
*V*_a_: *V*_applied_
UHV: ultrahigh vacuum
MIS: metal–insulator-semiconductor
VLS: vapor–liquid–solid
SED: secondary electron detector
