# Supplementary material for: Impact of the Tip-to-Semiconductor Contact in the Electrical Characterization of Nanowires
Source: ACS Omega. 2024 Jan 24;9(5):5788–97. doi: 10.1021/acsomega.3c08729 (PMC10851377; doi:10.1021/acsomega.3c08729)
Supplement: Supplementary file 1 — ao3c08729_si_001.pdf [file ao3c08729_si_001.pdf]

Supporting Information

Impact of the Tip-to-Semiconductor Contact in the Electrical Characterization of Nanowires

*Juliane Koch[1], Lisa Liborius[2], Peter Kleinschmidt[1], Werner Prost[2], Nils Weimann[2] and Thomas Hannappel[1]**

[1] Department of Mathematics and Natural Science, Institute for Physics, Fundamentals of Energy Materials, Ilmenau University of Technology, Ilmenau 98693, Germany

[2] Components for High Frequency Electronics (BHE), University of Duisburg-Essen, Duisburg 47057, Germany

The simulation done by LTSpice used the following parameters:

Duration of measurement: 120 s

PWL(0 0 30 6 60 0 90 -6 120 0)

Diode 1: I_s=6e-12 A; N=55; b_v=0.0005; I_{bv}=2e-25 A

Diode 2: I_s=5e-12 A; N=0.05; b_v= 0.5; I_{bv}=2e-25 A

Diode 3: I_s=5e-12 A; N=19; b_v= 0.1; I_{bv}=5e-15 A

Capacity 1: 1 pF

Capacity 2: 1 pF

Capacity 3: 800 pF

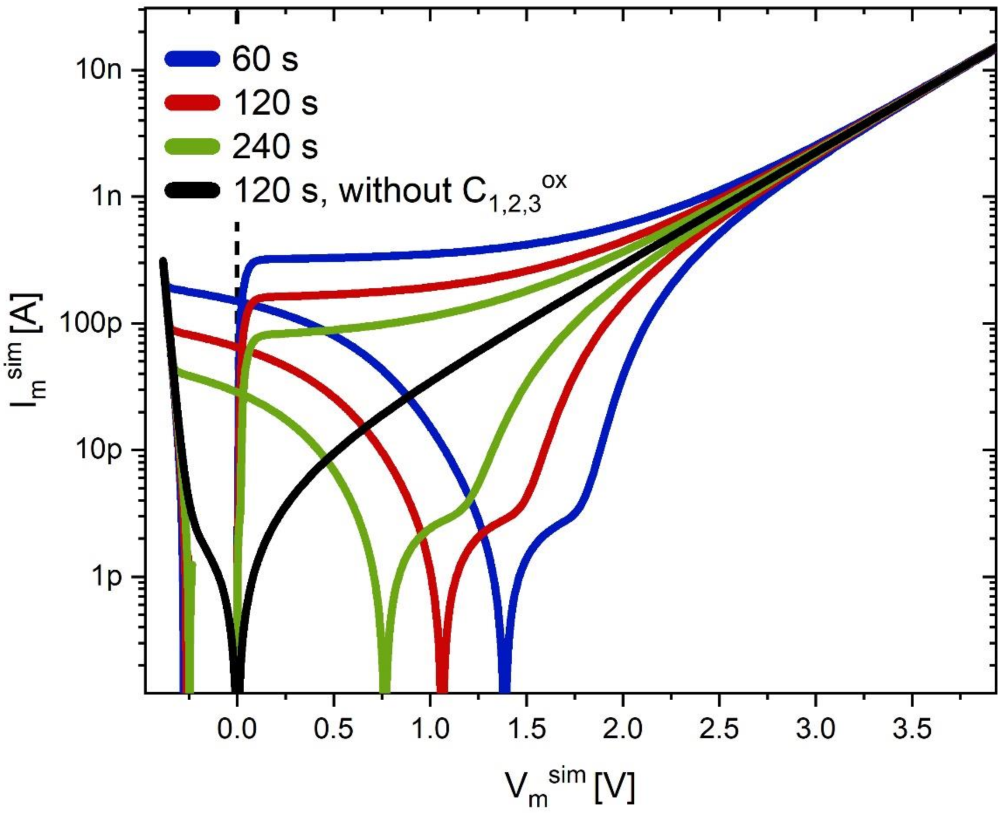

Figure S1. The simulation was also performed with different timescales as well as without $c_{1,2,3}^{ox}$. The results are in good agreement with the experimental recorded tendencies. A hysteresis appears, which is dependent on the timescale of the measurement Without $c_{1,2,3}^{ox}$, the is no hysteresis appearing. However, it must be considered that a local removal of the oxide layer and thus a general change in contact behavior can also be expected to lead to changes in the individual diode parameters, which have not yet been considered in the simulation.
